# Deep freezing to maintain the freshness of pork loin during long-term storage

**DOI:** 10.1007/s10068-021-00896-x

**Published:** 2021-05-13

**Authors:** SangYoon Lee, Eun Jeong Kim, Dong Hyeon Park, Yu Ra Ji, Guhyun Kang, Mi-Jung Choi

**Affiliations:** 1 Department of Food Science and Biotechnology of Animal Resources, Konkuk University, 120 Neungdong-ro, Gwangjin-gu, Seoul, 05029 Korea; 2 Refrigerator Research of Engineering Division, Home Appliance and Air Solution Company, LG Electronics, Changwon, Korea

**Keywords:** Pork loin, Freshness, Deep-freezing storage, Long-term storage

## Abstract

As storage temperature impacts frozen meat quality, we evaluated the ideal freezing and storage temperatures for pork loin, and effects of long-term storage at − 60, − 50, and − 18 °C on pork loin physicochemical properties. Pork loin was cut into 30 × 30 × 30 mm (50 g) and packed in air-containing box. Thereafter, they were stored at different freezing temperature for 6 months. Frozen pork loins were thawed at 4 °C. Samples frozen at − 18 °C exhibited surface dehydration (at 3 months) and high moisture loss surface dehydration-induced discoloration and toughening. However, samples frozen by deep freezing temperature (− 60 and − 50 °C) had lower values of thawing loss, WHC, and shear force than those of frozen at − 18 °C. Samples frozen at − 60 and − 50 °C maintained their freshness better than those frozen at − 18 °C; samples stored at − 60 °C showed significantly lower VBN than those stored at − 50 °C. Therefore, − 60 °C is suitable for freezing pork loins.

## Introduction

Freezing is used to prolong the shelf life of meat (Choi et al., 2016; Kim et al., 2015). Conventional freezing methods typically have a low freezing rate—occurring over an extended period—which is closely related to the final quality of meat products (Choi et al., 2016). Low freezing rates result in the formation of large, irregular ice crystals, which damage the cell membranes and muscle tissue (Kaale et al., 2013). These problems may enhance thawing loss, protein denaturation, and reduce the space within the myofibrils (Zhan et al., 2017).

To overcome these issues, various types of novel freezing techniques have been employed to accelerate the freezing rate (Zhan et al., 2017). Deep freezing shortens the freezing time, particularly the phase-transition time of water to ice, with extremely low temperatures (Choi et al., 2018). It also induces the formation of smaller ice crystals and minimizes tissue damage relative to that observed on using conventional freezing techniques (Soyer et al., 2010). Furthermore, the short phase-transition time of water to ice is crucial for inhibiting various aspects associated with quality loss, including protein denaturation and membrane breakage (Li and Sun, 2002).

The final temperature in the freezing process and the storage temperature also play a critical role in determining the amount of unfrozen water in a piece of meat (Leygonie et al., 2012). Biochemical reactions can occur in frozen meat at the temperature higher than − 20 °C, due to the presence of unfrozen water (Kim et al., 2020). This residual unfrozen water can initiate primary lipid oxidation in frozen meat, followed by radical secondary lipid oxidation, which results in unfavorable changes in color odor and flavors (Bellés et al., 2017). Estévez (2011) suggests that the optimal freezing storage temperature of meat products is − 40 °C. Zhou et al. (2010) recommend storing food at − 55 °C, with the goal of minimizing enzymatic reactions, oxidation, and recrystallization.

Despite the numerous advantages of freezing foods at extremely low temperatures, controversy exists regarding the effectiveness of ultra-low-temperature storage. It has been suggested that lowering the storage temperature to below the glass-transition temperatures of each food would lead to increased stability (Tolstorebrov et al., 2016). However, Farouk et al. (2003) contend that storing meat at <− 18 °C has little or no benefit with respect to the quality of frozen beef and is commercially useless. Furthermore, Grujić et al. (1993) observed that storing beef at an ultra-low temperature (<− 70 °C)—lower than the eutectic point of salts—was disadvantageous compared to a higher storage temperature due to higher drip loss. Choi et al. (2018) also suggested that storing lamb meat at − 60 °C was preferable to storing it at − 80 °C—when considering cost—though storing at − 80 °C produced slightly better meat quality. Optimal storage temperature seems to depend on the type of meat (when frozen meat quality and cost are both considered).

In this study, pork loin was chosen as this meat is liked by the general public. Pork loins were frozen and stored at conventional freezing temperature (− 18 °C) and deep-freezing temperatures (− 60 and − 50 °C), chosen based on the report by Zhou et al. (2010), which recommends storing foods at − 55 °C. The effect of deep freezing on meat quality was investigated to identify the optimal temperature for freezing and long-term storage. Temperature-induced changes in the physicochemical properties of the pork loin were observed and analyzed during a storage period of 6 months at freezing temperatures.

## Materials and methods

### Sample preparation

Pork loins were purchased at 48 h post mortem from a local butcher shop and cut into a 30 × 30 × 30 (width × length × height) mm cubes and packed in air-containing packages. After packaging, the pork loins were placed in freezers set at freezing temperature (− 60, − 50, or − 18 °C), and frozen until the temperature at the center of the samples reached − 60 °C, − 50 °C, or − 18 °C. During freezing, the temperature change was measured with T-type thermocouples placed at the center of each sample. The central temperature of pork loin samples was recorded during freezing and thawing using a data logger (Data Acquisition-MX 100, Yokogawa, Tokyo, Japan). After that, the samples were stored for 6 months in freezers (A255WD, LG Electronics, Seoul, Korea) and thawed in a refrigerator (A255WD) at 2 °C until the central temperature of the samples reached 0 °C. For comparison, 0-month samples were thawed after 24 h of freezing. The physicochemical properties of samples were evaluated after thawing.

### Thawing loss

Fresh pork loins were weighed before packaging, and thawed pork loins were weighed after removing the exudate of the samples using dry tissues. Thawing losses of samples were determined as the difference in the weight of fresh samples and thawed samples, as expressed by the following formula:

$$Thawing\,loss\,(\%) = \frac{Weight\,of\,fresh\,samples\,\left({{\rm g}}\right)-Weight\,of\,thawed\,sample\,\left({{\rm g}}\right)}{Weight\,of\,fresh\,sample \,\left({\rm g}\right)} \times 100.$$

### Water holding capacity (WHC)

Samples were thawed, and 1 g of each sample was wrapped in absorbent cotton and placed in centrifugal tube. The tubes containing the samples were centrifuged using a centrifuge separator (1736R, Labogene, Seoul, Korea) at 3000×*g* for 10 min at 4 °C. Samples were then weighed. The WHC of the pork loin was presented as the ratio of sample weight after centrifugation to sample weight before centrifugation, using the following formula:

$${W}HC (\%) = \frac{Weight\,of\,sample\,after\,centrifugation\,\left({{\rm g}}\right)}{Weight\,of\,sample\,before\,centrifugation\,\left({{\rm g}}\right)} \times 100.$$

### Shear force

Samples were cut into 10 × 20 × 10 (width × length × height) mm cubes. Shear force was measured using a texture analyzer (CT3 texture analyzer, Brookfield Engineering Labs Inc., Middleboro, MA, USA), and the parameters were set as: compression type, TA22 probe, TA/SBA fixture, test speed 2.5 mm/s, trigger load 5 g and target value 10 mm.

### Color

The lightness (CIE (Commission Internationale de l’Eclairag) L*), redness (CIE a*), and yellowness (CIE b*) values of the samples were measured using a colorimeter (CR-400 Chroma Meter, Konica Minolta Sensing Inc., Osaka, Japan) after calibrating with a white standard-plate (CIE L* = 96.79, CIE a* =  + 0.30, CIE b* =  + 1.67). Total color difference (*ΔE*) was calculated using the following equation that uses the differences between fresh and thawed samples:

$$\varDelta E=\sqrt{(\varDelta \mathrm{CIE }\,{L}^{*}{)}^{2}+(\varDelta \mathrm{CIE }\,{a}^{*}{)}^{2}+(\varDelta \,\mathrm{CIE }{b}^{*}{)}^{2}}.$$

### Volatile basic nitrogen (VBN)

Volatile basic nitrogen (VBN) of pork loin was determined using Conway’s micro-diffusion method (Conway, 1950). Sample (5 g) was homogenized with 45 mL of distilled water by slap-type homogenizer (WS-400, Shanghai Zhisun Equipment Co. Ltd., Shanghai, China) for 180 s. The homogenate was filtered using Whatman No.1 filter paper (GE Healthcare Life Science, Sheffield, UK). After filtering, 1 mL of filtrate was added to the outer side of the Conway dish. A mixture of 1 mL of 0.01 N H_3_BO_3_ and 0.1 mL of Conway solution (mixture of 0.066% methyl red in ethanol and 0.066% bromocresol green in ethanol) was added dropwise into the inner side of the Conway dish. Afterward, 1 mL of 50% K_2_CO_3_ was added to the outer side of the Conway dish. The Conway dish was closed and placed in an incubator (HB-103 M, Vision Lab & Instrument, Incheon, Korea) at 37 °C for 2 h. After incubation, the sample was titrated using 0.02 N H_2_SO_4_ until the Conway reagent became red in color. VBN contents were determined after adding 0.02 N H_2_SO_4_ on the inner side of the Conway dish. The VBN values were calculated using the following formula:

$$VBN (\mathrm{mg}\%/100 \mathrm{g})= \frac{14.007 \times (a - b) \times f \times 100 \times c}{ S}$$

where *a* and *b* are the titration volumes (mL) for the sample and the blank, respectively, f is the factor H_2_SO_4_, *S* is the weight (g) of the sample, and *c* is the dilution factor.

### Thiobarbituric acid reactive substance (TBARS)

The TBARS of each sample was measured using the procedure proposed by Choi et al. (2018). Sample (5 g) was homogenized using 45 mL of distilled water for 60 s. The homogenate was filtered using a Whatman No.1 filter paper (GE Healthcare Life Science). The filtrate (0.5 mL) was added to 4.5 mL TBA solution (0.25 N HCl, 15% trichloroacetic acid, and 0.375% TBA reagent) in a test tube. The tube was heated at 95 °C for 15 min in a water bath (BF-30SB), cooled at 20–25 °C. Afterward, the tube was centrifuged at 3000×*g* at 4 °C for 10 min. The 200 μL of the supernatant was added in a 96-well plate (SPL Life Science, Pocheon, Korea), and absorbance was measured using a spectrophotometer (MultiskanTM GO UV/VIS, Thermo Fisher, Waltham, MA, USA) at 535 nm. The TBA value of the samples is expressed as mg MDA/kg (mg malodinaldehyde/kg).

### Statistical analysis

All results were expressed as mean ± standard deviation (SD). To analyze interaction effects of component content changes in response to storage temperature used for deep-freezing (− 60 and − 50 °C), data were analyzed using two-way ANOVA. In cases in which one-way ANOVA revealed significant differences (*p* < 0.05), a post-hoc test was conducted using Duncan’s multiple-range test. All statistical analyses were performed using SPSS software 24.0 (SPSS Inc., Chicago, IL, USA).

## Results and discussion

### Freezing and thawing curves

**Fig. 1:**
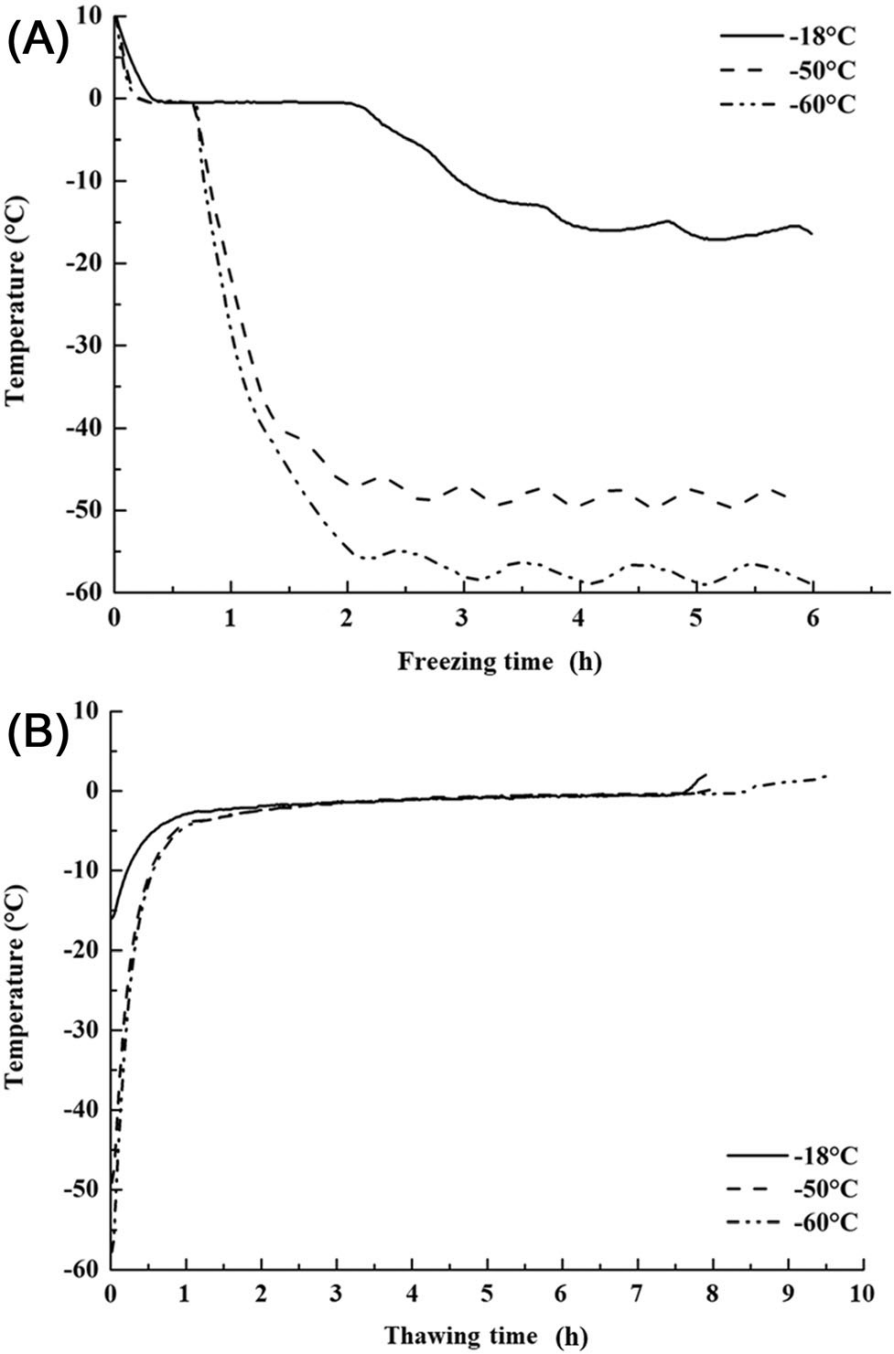
Freezing curves (**A**) and thawing curves (**B**) of pork loins at various freezing temperatures (0 months)

Figure 1 shows the temperature–time profiles of pork loin during freezing and thawing. The phase-transition time was defined as the time required to pass through the range of − 5 to − 0.5 °C, the maximal ice-crystal-forming temperature band in this study. The phase-transition time of pork loin frozen at − 18 °C was about 2 h, whereas phase-transition was completed within 0.5 h in pork loins frozen at − 60 and − 50 °C (Fig. 1A).

Freezing was divided into a pre-cooling stage (− 1 to 4 °C), a phase-transition stage (− 6 or − 5 to − 1 °C), and a sub-cooling stage (less than − 6 or − 5 °C) (Zhang et al., 2018). Most water crystallizes in the phase-transition stage, which could dramatically influence frozen food quality (Li and Sun, 2002). Consequently, passing the maximal ice crystal forming band quickly could result in the formation of smaller ice crystals, thereby alleviating the deterioration of muscle fibers and meat quality (Kaale et al., 2013; Zhang et al., 2018). According to Choi et al. (2018), broadly defined quick freezing as a phase-transition time of < 30 min. Under this criterion, freezing pork loins at − 60 and − 50 °C was classified as quick freezing, which was presumed to result in the formation of smaller ice crystals than freezing at − 18 °C, which was categorized as slow freezing.

For the thawing process (Fig. 1B), the phase-transition times of pork loins frozen at − 60, − 50 and − 18 °C were about 7 h. Kim et al. (2015) have described the manner in which freezing methods influence thawing time. Thawing times of pork loins are thought to be aggregated into a certain time range regardless of the various freezing temperatures used in this study. Choi et al. (2018) observed no distinct difference in the phase-transition time when thawing lamb meat stored at various temperatures, which is consistent with our result.

### Thawing loss, WHC, and shear force

Table 1 presents the thawing loss of pork loin with different freezing temperatures and storage periods. Thawing losses of all samples were about 4% after 0-month freezing storage. However, thawing losses of samples frozen at − 18 °C gradually increased with storage period, whereas those of samples frozen at − 60 and − 50 °C were not significantly different at up to 6 months of storage (*p* > 0.05). Thawing losses of samples frozen at − 18 °C were the highest among all storage periods except for 0-month (*p* < 0.05) and reached 13.92% after 6 months of storage. Samples frozen at − 60 and − 50 °C maintained a thawing loss of about 5% after 6 months of storage.

**Table 1:** Changes in the thawing loss and WHC of the pork loins depending on the various freezing temperatures and storage periods

Storage period (months)	− 18	− 50	− 60
*Storage temperature (°C)*			
Drip loss (%)			
0	3.89 ± 0.54^cA^	4.07 ± 1.21^abA^	4.15 ± 0.21^abA^
1	5.94 ± 1.04^cA^	4.23 ± 1.30^abB^	3.76 ± 0.82^bB^
3	8.56 ± 2.07^bA^	3.08 ± 0.60^bB^	3.43 ± 0.38^bB^
6	13.92 ± 1.80^aA^	4.75 ± 0.87^aB^	5.28 ± 1.07^aB^
WHC (%)			
Control	84.06 ± 2.94^a^	84.06 ± 2.94^a^	84.06 ± 2.94^a^
0	79.95 ± 2.68^bA^	76.21 ± 2.24^bA^	79.52 ± 1.88^bA^
1	72.33 ± 3.13^cB^	80.97 ± 3.95^aA^	80.36 ± 2.67^abA^
3	69.23 ± 0.95^cdB^	75.01 ± 1.79^bA^	76.91 ± 2.93^bcA^
6	66.76 ± 1.40^ dB^	73.78 ± 3.14^bA^	73.24 ± 0.68^cA^
Shear force (N)			
Control	38.44 ± 2.34^c^	38.44 ± 2.34^a^	38.44 ± 2.34^a^
0	34.85 ± 3.75^cdB^	39.92 ± 2.01^aA^	38.97 ± 2.52^aA^
1	30.42 ± 3.32^ dB^	36.70 ± 3.74^aA^	36.82 ± 3.91^aA^
3	51.18 ± 5.02^bA^	38.78 ± 5.75^aB^	39.08 ± 3.20^aB^
6	56.57 ± 3.14^aA^	39.65 ± 3.71^aB^	37.81 ± 3.40^aB^

^a−d^Means significantly different within the same storage temperature (*p* < 0.05)

^A,B^Means significantly different within the same storage period (*p* < 0.05)

The amount of exudate generated during freezing and thawing is considered a parameter that represents the quality of frozen meat (Leygonie et al., 2012). Thawing loss implies nutrient loss in the form of water-soluble and sarcoplasmic proteins (Huff-Lonergan and Sosnicki, 2003). Freezing is relevant to the size and distribution of ice crystals formed during the freezing process (Añón and Calvelo, 1980). Freezing rate is also associated with thawing loss in thawed meat (Hong et al., 2005; Leygonie et al., 2012) and is regarded as an indicator for measuring the damage caused to the muscle structure by the freezing procedure (Kondratowicz et al., 2006). Lambert et al. (2001) described thawing loss as the effluence of intercellular compounds from rupture of the fiber membrane induced by ice crystals. In addition, many studies have analyzed factors affecting thawing loss, such as post-mortem aging, frozen storage, and thawing conditions (Añón and Calvelo, 1980; Kondratowicz et al., 2006). In this study, thawing loss of pork loin frozen at − 18 °C was higher than in samples frozen at − 60 and − 50 °C; this result was similar to the findings of Zhang and Ertbjerg (2019), which showed that slow freezing induced more thawing loss in pork than fast freezing due to protein denaturation, resulting in water loss in myofibrillar proteins. At deep-freezing storage temperatures, there was no significant difference between storage periods, with about 0.5% difference in thawing losses between two storage temperatures after 1 month.

The WHC of pork loin with different freezing temperatures and storage periods (Table 1). The WHC of fresh pork loin was 84%, and it decreased stepwise with storage periods. In comparison to control and 0-month samples, the WHC of frozen and deep-frozen samples was significantly lowered by the freezing and thawing procedure. After 1 month of storage, the WHC of samples frozen at − 18 °C was the lowest value of all storage periods (*p* < 0.05), whereas those of samples frozen at − 50 and − 60 °C were not significantly different (*p* > 0.05). The WHC of samples frozen at − 18 °C was below 70% after 3 months of storage, although values for samples frozen at − 60 and − 50 °C were above 73% after 6 months of storage.

WHC is the ability of the muscle to reserve water from external forces such as gravity and heating (Huff-Lonergan and Sosnicki, 2003). Low-WHC meat usually leads to low-quality processed products (Huff-Lonergan and Sosnicki, 2003). It is known that freezing, low-temperature storage, and thawing processes reduce the WHC of meat (Añón and Calvelo, 1980; Vieira et al., 2009). Myofibrils comprising about 82–87% of a muscle cell hold about 85% of water by capillary force. In this way, the loss of WHC is associated with the destruction of muscle fiber structures and protein denaturation and/or modification, which shrink myofibrils (Huff-Lonergan and Sosnicki, 2003). For this reason, ice crystals formed during the freezing process induce the destruction of cell membrane and concentration of solutes by several fold in muscle cells, lowering the WHC of meat (Huff-Lonergan and Sosnicki, 2003). In this study, freezing and thawing processes markedly affected the WHC of pork loins. In particular, pork loins frozen at − 18 °C had lower WHC than did those frozen at − 60 and − 50 °C. It is thought that pork loins frozen at − 18 °C formed larger ice crystals during freezing. Comparing WHC values of samples frozen at − 18 °C, we found that WHC declined 7% from 0 to 1 month, whereas only a 3% decrease was observed from 1 to 3 months of storage. It was assumed that freezing procedure was the major factor affecting WHC of pork loin, rather than deterioration during freezing storage, such as that caused by surface dehydration. There was no significant difference in WHC of pork loins between − 60 and − 50 °C in any storage period (*p* > 0.05).

The shear force of thawed pork loin with various freezing temperatures and storage periods (Table 1). The shear force of fresh pork loin was 38.44 N, and it was affected by storage temperatures. The shear force of samples frozen at − 18 °C decreased to 30.42 N after 1 month of storage, the lowest value measured for this sample. However, this value rebounded to above 50 N after 3 months of storage, which was the highest value in any storage period (*p* < 0.05). After 6 months of storage, the shear force of samples frozen at − 18 °C was 56.57 N, which was the highest among all storage temperatures and storage periods. In contrast, the shear force of samples frozen at − 60 and − 50 °C maintained its initial value for up to 6 months of storage without any significant difference (*p* > 0.05).

Lagerstedt et al. (2008) reported that the pork meat shear force decreased after freezing and thawing. This has been known to occur through the loss of cell membrane durability caused by ice crystal formation and decreased shear force (Lui et al., 2010). Leygonie et al. (2012) also reported that the loss of physical structure induced by ice crystal formation caused the tenderization of meat. These ice crystals, formed during freezing, destroy myofibrils and expand intercellular spaces (Vieira et al., 2009). Lagerstedt et al. (2008) also found that the shear force of meat treated by freezing is related to the storage period and storing conditions. In this study, ice crystal formation and the loss of structure might have affected samples frozen at − 18 °C, showing a decreasing tendency for up to 1 month. In addition, unexpected increasing shear force was observed in the sample frozen at − 18 °C after 3 months of storage. This was attributed to cellular shrinkage and toughening caused by surface dehydration (Drummond and Sun, 2010). On the other hand, pork loins frozen at − 60 and − 50 °C maintained the shear force of fresh pork loin during freezing storage. Between the two lower storage temperatures, shear forces of pork loins frozen at − 60 °C were more similar to control values than those of pork loins frozen at − 50 °C in whole storage periods. However, no significant difference was observed in shear force values between deep-freezing storage periods (*p* > 0.05).

### Color and appearance

Table 2 shows the color parameters and total color differences of pork loin with different freezing temperatures and storage periods. The CIE L* value of fresh pork loin was 43.36, and storage temperature significantly affected CIE L* values. Those of samples frozen at − 60 and − 50 °C fluctuated in the range of 43–47 but were not significantly different between samples stored at these two temperatures in all storage periods (*p* > 0.05). Meanwhile, CIE L* values of pork loins frozen at − 18 °C slightly declined up to 1 month of storage and markedly increased above 50 after 3 months of storage. The CIE L* value of pork loin frozen at − 18 °C was above 56 in month 6 of storage. The CIE b* values showed a trend similar to the CIE L* value. CIE b* of fresh pork loin was 3.73, and samples frozen at − 60 and − 50 °C had similar CIE b* values in all storage periods (*p* > 0.05). Those values were shifted in the range of 3.5–4.6. The CIE b* values of samples frozen at − 18 °C also rose notably after 3 months of storage (*p* < 0.05). In month 6, the CIE b* value of pork loin frozen at − 18 °C was over 10, which was the highest value in all storage temperatures and periods. The CIE a* values of pork loins had no such obvious trend. All CIE a* values were in the range of 4.5–6.5. In this way, total color difference (*ΔE*) of pork loin frozen at − 18 °C was strikingly increased after 3 months of storage (*p* < 0.05) due to the enormously high CIE L* and CIE b* values.

**Table 2:** Changes in the color of the pork loins depending on the various freezing temperatures and storage periods

Storage temperature (°C)	Color				
	Storage period (months)				
	Control	0	1	3	6
CIE L*					
− 18	43.36 ± 1.37^c^	41.94 ± 1.45^cdB^	41.27 ± 1.66^ dB^	53.88 ± 2.15^bA^	56.08 ± 1.75^aA^
− 50	43.36 ± 1.37^b^	44.06 ± 0.51^bA^	43.75 ± 1.52^bA^	47.01 ± 1.75^aB^	43.71 ± 2.27^bB^
− 60	43.36 ± 1.37^b^	44.65 ± 1.45^abA^	44.51 ± 1.60^abA^	45.65 ± 2.10^aB^	44.83 ± 2.24^abB^
CIE a*					
− 18	6.03 ± 0.63^a^	5.82 ± 0.46^aA^	5.75 ± 0.53^aA^	4.53 ± 0.73^bB^	6.49 ± 0.81^aA^
− 50	6.03 ± 0.63^a^	5.49 ± 0.54^abA^	4.85 ± 0.55^bB^	5.48 ± 1.13^abA^	5.06 ± 0.52^bB^
− 60	6.03 ± 0.63^a^	5.26 ± 0.71^abA^	5.30 ± 1.12^abAB^	5.63 ± 0.49^abA^	4.90 ± 0.36^bB^
CIE b*					
− 18	3.73 ± 0.98^c^	3.68 ± 0.69^cA^	4.00 ± 0.46^cAB^	6.65 ± 0.90^bA^	10.19 ± 0.30^aA^
− 50	3.73 ± 0.98^a^	4.07 ± 0.30^aA^	3.52 ± 0.93^aB^	4.54 ± 0.79^aB^	3.88 ± 0.97^aB^
− 60	3.73 ± 0.98^a^	3.97 ± 0.48^aA^	4.45 ± 0.79^aA^	4.13 ± 0.58^aB^	3.87 ± 0.31^aB^
*ΔE*					
− 18		1.75 ± 1.22^cA^	2.30 ± 1.53^cA^	11.12 ± 1.39^bA^	14.32 ± 1.55^aA^
− 50		1.10 ± 0.49^bA^	1.98 ± 1.88^bA^	4.10 ± 1.46^aB^	2.30 ± 1.21^bB^
− 60		1.89 ± 1.19^aA^	2.38 ± 0.68^aA^	2.81 ± 1.52^aB^	2.67 ± 0.99^aB^

^a–d^Means significantly different within the same storage temperature (*p* < 0.05)

^A,B^Means significantly different within the same storage period (*p* < 0.05)

**Fig. 2:**
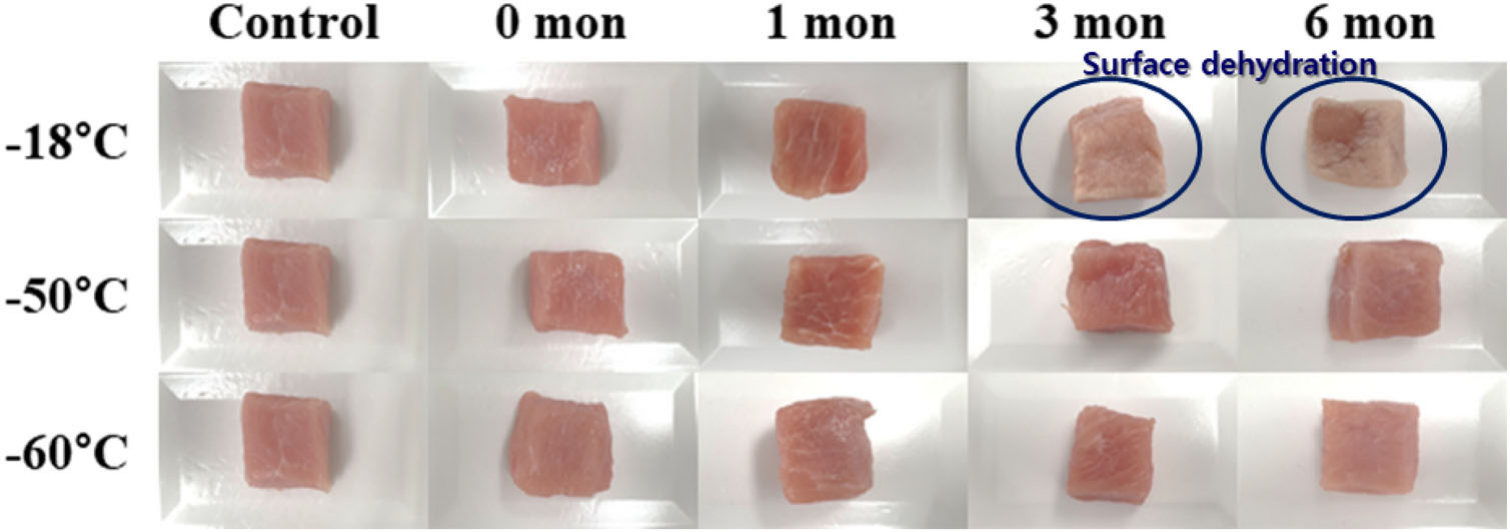
Visible appearance of the pork loins depending on the various freezing temperatures and storage periods

There are many studies indicating that freezing rates do not affect the color of meat (Zhang et al., 2018). Kim et al. (2015) reported that color of pork did not present any significant difference with different freezing and thawing treatments. This phenomenon was elucidated by Fernández et al. (2007) who ascribed the reason to recovering myoglobin conformation after thawing treatment. Various factors affecting the color of frozen meat have been reported. In slow freezing, higher amounts of thaw drip may lead to increased light reflection, resulting in lighter color (Muela et al., 2010). Muela et al. (2015) suggested that the increase of CIE b* is a result of denaturation of myoglobin and metmyoglobin, and lipid oxidation with storage period. Meats affected by surface dehydration (freezer burn) during freezing storage could have a darker or light-brown color (Drummond and Sun, 2010). Based on these results, we infer that the increment in CIE L* and CIE b* values in pork loins frozen at − 18 °C was due to surface dehydration (Fig. 2). After 3 months of storage, these values increased compared to those observed in case of other storage temperatures and earlier storage periods, resulting in an increase in *ΔE* values. In contrast, pork loins frozen at − 60 and − 50 °C did not show any distinct change in color. Notably, the *ΔE* value of pork loins frozen at − 50 °C and stored for 3 months was significantly higher, though there was no significant difference between the *ΔE* values at the two storage temperatures, − 60 and − 50 °C. This supports the findings of Kim et al. (2015) and may be attributed to the recovery of myoglobin conformation, as stated by Fernández et al. (2007) as well as to the interfering factors affecting the color of frozen meat, such as myoglobin denaturation and surface dehydration in response to lower storage temperatures.

### TBARS and VBN

Table 3 shows the TBARS of pork loin with various freezing temperatures and storage periods. The TBARS value of fresh pork loin was 0.2090 mg MDA/kg, while that the samples fluctuated in the range 0.1870–0.2627 mg MDA/kg. After 6 months of storage, the TBARS values of frozen pork loins were not significantly different—for all storage temperatures—compared to those of fresh pork loin. Additionally, no significantly different TBARS values were observed for frozen pork loins among all storage temperatures and across all storage periods.

**Table 3:** Changes in the VBN and TBARS of the pork loins depending on the various freezing temperatures and storage periods

Storage period (months)	− 18	− 50	− 60
*Storage temperature (°C)*			
TBARS (mg MDA/kg)			
Control	0.2090 ± 0.0094^b^	0.2090 ± 0.0094a	0.2090 ± 0.0094^a^
0	0.2001 ± 0.0056^bA^	0.1919 ± 0.0098^aA^	0.1870 ± 0.0138^bA^
1	0.2627 ± 0.0578^aA^	0.2114 ± 0.0217^aA^	0.2090 ± 0.0113^aA^
3	0.2090 ± 0.0098^bA^	0.2017 ± 0.0342^aA^	0.1883 ± 0.0128^bA^
6	0.2302 ± 0.0252^abA^	0.2180 ± 0.0244^aA^	0.1960 ± 0.0056^abA^
VBN (mg/100 g)			
Control	6.37 ± 0.29^d^	6.37 ± 0.29^c^	6.37 ± 0.29^c^
0	6.34 ± 0.20^dA^	6.37 ± 0.18^cA^	6.31 ± 0.25^cA^
1	6.77 ± 0.18^cA^	6.52 ± 0.15^cB^	6.49 ± 0.20^cB^
3	8.34 ± 0.25^bA^	7.41 ± 0.31^bB^	7.19 ± 0.24^bB^
6	9.26 ± 0.31^aA^	8.57 ± 0.14^aB^	8.33 ± 0.14^aB^

^a−d^Means significantly different within the same storage temperature (*p* < 0.05)

^A,B^Means significantly different within the same storage period (*p* < 0.05)

Lipid oxidation results in deteriorated sensory properties associated with meat. One possible explanation for lipid oxidation is the aldehyde reaction with other compounds, resulting in the production of substances that do not react with thiobarbituric acid (De las Heras et al., 2003). Another possible explanation is that the TBARS values manifest a typical ‘induction-propagation-termination’ cycle that causes the increase/decrease pattern (Barnett et al., 1991). Thus, both time and temperature are major factors, as lipid oxidation is a complex process consisting of multiple reactions. Medić et al. (2018) reported that the TBARS of pork ham, loin, and rib frozen at − 18 °C was maintained below 0.18 mg MDA/kg up to 6 months. In this study, it was thought that pork loins were oxidatively stable during storage at freezing temperatures, based on the TBARS values of all samples—that did not exceed 1.0 mg MDA/kg—which means the standard of rancid odor and flavor of meat products (Kolsarıcı et al., 2010).

Table 3 shows the VBN of pork loin with various freezing temperatures and storage periods. VBN of fresh pork loin was 6.37 mg/100 g, and freezing treatment seemed not to affect the VBN content. The VBN contents of frozen pork loin were not different from that of fresh pork loin at 0 months (*p* > 0.05). After 1 month, VBN contents of samples varied by storage temperature. Those of samples frozen at − 18 °C were the highest in each storage period (*p* < 0.05), whereas samples frozen at − 60 and − 50 °C were not significantly different in all storage periods. After 6 months of storage, the VBN content of pork loin frozen at − 18 °C was 9.26 mg/100 g, and those of pork loin frozen at − 60 and − 50 °C were 8.44 and 8.57 mg/100 g, respectively.

Meat proteins disintegrate into nitrogen-containing compounds (ammonia and amines) that are used to estimate the VBN content via proteolytic activities of enzymes and bacteria (Kim et al., 1998). Kim et al. (1998) showed that VBN in frozen pork is generated by the decomposition of ATP by ATPase. Reactions related to increasing the amount of VBN can be halted by storing at temperatures below the glass transition temperature (generally below − 70 °C) (Tolstorebrov et al., 2016). Such low temperatures are significant for stabilizing the proteins in frozen fish. In addition, studies have reported that the amount of VBN compounds increases during storage at freezing temperatures in pork due to the mechanical change of membranes and myofibrillar proteins (Kim et al., 1998; Zhang and Ertbjerg, 2019). Myofibrils are affected by protein denaturation, this results in increased surface hydrophobicity and unfolding that lead to water loss and dissociation of myosin molecules. Microbial spoilage is known to be inhibited during freezing and storage at freezing temperatures (Leygonie et al., 2012). In this way, microbial proteolytic activity can be curbed or stopped; we found that freezing and storing the pork loin at − 60 and − 50 °C could aid the maintenance of the VBN content during storage at freezing temperatures. In all storage periods, VBN contents of the pork loins frozen at − 60 °C were lower than those of pork loins frozen at − 50 °C. However, the difference between the VBN content in response to storage at various temperatures was not significant (*p* > 0.05).

### Two-way ANOVA

Table 4 shows the F-values of pork loin for the storage periods and storage temperatures during deep-freezing − 60 and − 50 °C). Thawing loss, WHC, CIE L*, *ΔE*, and VBN were affected by the storage period. In contrast, only VBN was affected by the storage temperature (*p* < 0.05). Shear force, CIE a*, CIE b*, and TBARS values were not affected by the storage periods or storage temperatures. In this way, storage at freezing temperatures could effectively maintain the quality of pork loin, especially as certain parameters such as shear force, CIE a*, CIE b*, and TBARS were not affected by the storage period or temperature. Additionally, parameters, such as thawing loss, WHC, CIE L* and *ΔE* were not noticeably different between samples stored at − 60 and − 50 °C. Only VBN was significantly changed in response to storage at − 60 and − 50 °C; this result contrasts with the results presented in Table 4, which show no significant difference between storage at − 60 and − 50 °C. Consequently, none of the parameters showed a significant change upon storage of samples at − 60 and − 50 °C, except VBN. Hence, − 60 °C was the optimal storage temperature for pork loin in this study.

**Table 4:** F-values for parameters of pork loins with respect to time (storage periods) and temperature (− 60 and − 50 °C)

Parameter	Time		Temperature		Time × temperature	
	F	*P*	F	*P*	F	*P*
Thawing loss	5.507	< 0.01	0.147	NS	0.526	NS
WHC	192.610	< 0.001	0.172	NS	0.149	NS
Shear force	1.102	NS	0.524	NS	0.105	NS
CIE L*	5.261	< 0.01	0.356	NS	1.502	NS
CIE a*	1.933	NS	0.077	NS	0.641	NS
CIE b*	1.023	NS	0.256	NS	2.070	NS
*ΔE*	6.992	< 0.001	0.049	NS	2.354	NS
VBN	199.136	< 0.001	4.223	< 0.05	0.680	NS
TBARS	2.364	NS	2.803	NS	0.484	NS

F, F-value; *P*, *p* value; WHC, Water holding capacity; *ΔE*, Total color difference; VBN, Volatile basic nitrogen; TBARS, Thiobarbituric acid-reactive substances; Time × Temperature, Influence by both storage periods and temperature; NS, Not significant

In conclusion, pork loins frozen at − 60 and − 50 °C—referred to as deep freezing—undergo quick freezing based on phase transition time. Generally, pork loins frozen at − 60 and − 50 °C maintained their quality, in that it was similar to that of fresh pork loin in terms of properties, such as thawing loss, shear force, and color; pork loins frozen at − 18 °C did not retain their freshness when these properties were considered. WHC and VBN content of the pork loins frozen at − 60 and − 50 °C were less altered compared to pork loins frozen at − 18 °C. Further, pork loins stored at − 60 °C had significantly lower VBN than those stored at − 50 °C. Thus, storage temperatures for deep-freezing could effectively influence the quality of the pork loin and could also extend their shelf-life. It is thought that freezing pork loins at − 60 and − 50 °C might result in the formation of smaller ice crystals as this process involves quick freezing, which is more advantageous for maintaining the quality during storage (compared to freezing the meat at − 18 °C). On considering the quality of the pork loins frozen at − 60 and those frozen at − 50 °C—particularly with respect to VBN—it seems that − 60 °C is the optimal freezing and storage temperature for pork loins.
